# Ethical dimensions of translational developmental neuroscience research in autism

**DOI:** 10.1111/jcpp.13494

**Published:** 2021-08-18

**Authors:** Arianna Manzini, Emily J.H. Jones, Tony Charman, Mayada Elsabbagh, Mark H. Johnson, Ilina Singh

**Affiliations:** 1 Centre for Ethics in Medicine, University of Bristol, Bristol, UK; 2 Radcliffe Department of Medicine, MRC Weatherall Institute of Molecular Medicine, University of Oxford, Oxford, UK; 3 Centre for Brain and Cognitive Development, Birkbeck, University of London, London, UK; 4 Institute of Psychiatry, Psychology & Neuroscience, King’s College London, London, UK; 5 Azrieli Centre for Autism Research, Montreal Neurological Institute-Hospital, McGill University, Montreal, Canada; 6 Research Institute of the McGill University Health Centre, Montreal, Canada; 7 Department of Psychology, University of Cambridge, UK; 8 Department of Psychiatry and Wellcome Centre for Ethics and Humanities, University of Oxford, Oxford, UK

**Keywords:** autism, biomarkers, genetics, neurodevelopment, infant siblings, ethics

## Abstract

**Background:**

Since the 1990s, increasing research has been devoted to the identification of biomarkers for autism to help attain more objective diagnosis; enable early prediction of prognosis; and guide individualised intervention options. Early studies focused on the identification of genetic variants associated with autism, but more recently, research has expanded to investigate neurodevelopmental markers. While ethicists have extensively discussed issues around advances in autism genomics, much less ethical scrutiny has focused on research on early neurodevelopment and on the interventions being developed as a result.

**Objectives:**

We summarise the current state of the science on the identification of early markers for autism and its potential clinical applications, before providing an overview of the ethical issues arising from increasing understanding of children’s neurodevelopment in very early life.

**Results:**

Advances in the understanding of brain and behavioural trajectories preceding later autism diagnosis raise ethical concerns around three themes: (1) New models for understanding autism; (2) Risks and benefits of early identification and intervention; and (3) Communication of early concerns to families. These ethical issues should be further investigated in research conducted in partnership with autistic people and their families.

**Conclusions:**

This paper highlights the need for ethical scrutiny of early neurodevelopmental research in autism. Scrutiny requires expertise and methods from the basic sciences and bioethics, as well as constructive collaborations among autistic people, their parents, and autism researchers to anticipate early interventions that serve the community’s interests and accommodate the varied experiences and preferences of people on the spectrum and their families.

## 1 Introduction

Autism can be viewed as a neurodevelopmental condition characterised by difficulties in social communication, patterns of restricted and repetitive behaviour and sensory anomalies (American Psychiatric Association, 2013); and/or as a fundamental part of someone’s identity that comes with strengths and weaknesses and is part of the spectrum of human neurodiversity (Kapp, 2020). Diagnosis of autism occurs in ~1-2% of children, and diagnosed children commonly have co-occurring mental health and medical conditions. In many, but not all diagnosed cases, autism emerges in the early years (Lord et al., 2020). Since the 1990s, research has increasingly focused on the identification of biomarkers for autism. Biomarkers are measurable indicators of a biological state and they could be used to supplement an autism diagnosis based on behavioural criteria; indicate an individual’s increased likelihood of developing autism; identify individuals developing autism before they manifest clear observable traits; and provide indications for targeted treatments (Yusuf & Elsabbagh, 2015). While early studies focused on the potential for genetic biomarkers, research has more recently expanded to investigate other markers of neurodevelopment.

Neurodevelopmental markers are measures of infant brain or cognitive function that can be captured with non-invasive techniques like electroencephalography, eyetracking or near infrared spectroscopy and may predict the emergence of later autism or co-occurring traits. Neurodevelopmental markers may be closer than genetic markers to the final common pathway to autism symptoms, if autism is seen as an unfolding developmental process; and they might offer more information about both potential later outcomes on that process and the mechanisms through which developmental changes will occur, hence providing insights into how to support a child’s development.

Efforts to identify genetic and neurodevelopmental markers for autism (the latter termed ‘early autism research’ in this paper) are usually framed around supporting development to optimise outcomes. This framing raises critical ethical questions, not least because ‘optimal outcome’ has been defined in various (at times conflicting) ways in the autism literature and within the autism community^1^. Definitions have included: ceasing to meet autism diagnostic criteria at some point during development; preventing autism in infants with enhanced likelihood for autism; and developing skills (e.g., independence, making friends) that the individual and their family consider meaningful and desirable (Georgiades & Kasari, 2018).

Bioethicists and social scientists have extensively discussed ethical issues around advances in autism genomics in a body of literature known as ‘gen-ethics’ (see Hens, Peeters, & Dierickx, 2016). Empirical studies have been conducted to investigate the autism community’s attitudes towards tests to identify the genetic aetiology of an individual’s autism diagnosis (e.g., Reiff et al., 2015; Wagner et al., 2020), although this body of literature has important limitations (Yusuf & Elsabbagh, 2015), particularly an almost exclusive focus on parents’ attitudes, as compared to the views of autistic people (although these are not mutually exclusive categories). Much less ethics scrutiny has been given to research efforts on early brain and cognitive development in the autism field and on the interventions being developed as a result. A few studies have investigated the autism community’s perspectives in this area (MacDuffie et al., 2020), and issues concerning research priority setting (see Fletcher-Watson et al., 2017). This is an important gap because both similar and distinct ethical concerns to those related to autism genomic information may arise from increasing understanding of children’s neurodevelopment in very early life.

In this paper, we begin by summarising the current state of the science on the identification of biomarkers (both genetic and neurodevelopmental) for autism, and its clinical applications. We then provide an overview of the ethical issues arising from the identification of neurodevelopmental markers for autism, before making recommendations on how early autism research should be reshaped in focus and methods to address these concerns.

## 2 Research on autism biomarkers

### Advances in autism genomics

Estimated at 50-80%, the high heritability of autism indicates that a substantial proportion of variation in autistic symptoms is genetic (Tick et al., 2016). About 20-30% of cases of autism can be linked to rare genetic variants (often duplications/deletions) that have profound effects on development and can raise the likelihood of developing autism up to 40 fold (Dias & Walsh, 2020); some are associated with particular syndromes (e.g., Fragile X, tuberous sclerosis). The remaining majority of cases are associated with the accumulated effects of many thousands of common variations (Grove et al., 2019), and can be studied through polygenic ‘scores’, which summarise an individual’s autism-associated variants to operationalise individual genetic ‘load’. Thus, different genetic profiles associate with autism. However, rare and common variants interact in complex ways with each other and with environmental factors, and understanding of these interactions is currently limited.

### Neurobiological mechanisms and early brain development

Genetic alterations associated with autism provide insight into the neurobiological mechanisms that underpin the condition. Such work suggests important roles for synaptic function, activity-dependent transcription and translation, and neuroinflammation (de La Torre-Ubieta, Won, Stein, & Geschwind, 2016). These processes operate across the brain and do not readily map onto the specific regions or processes common to early ‘core deficit’ accounts of autism, such as the theory of mind account (Baron-Cohen, Leslie, & Frith, 1985), the weak central coherence account (Happé & Frith, 2006), and the social motivation hypothesis (Clements et al., 2018). While the peak profiles of expression of these genes occur in prenatal or very early postnatal development (see Parikshak et al., 2013), pinpointing this stage of development as a critical time window, overt behavioural autism symptoms generally do not appear until the end of the first year or later; parents typically express concerns around 16-18 months postnatal. To better understand how these alterations in early neurobiology contribute to autism, trajectories of brain development must be studied from early in infancy before behavioural symptoms emerge, as autistic people later experience many secondary difficulties (e.g., anxiety, depression, bullying, isolation) that have their own impacts on brain function, likely masking the causal processes that initially triggered early behavioural symptoms (Johnson, Charman, Pickles, & Jones, 2021).

Prospective longitudinal studies of infants with an elevated likelihood of developing autism allow us to map the very early changes in brain and cognitive development that precede the emergence of diagnostic symptoms (Wolff & Piven, 2020). The most common design is to study infants with an older sibling with autism (‘infant sibs’), who have about 20% chance of meeting autism criteria at age 3 (Ozonoff et al., 2011), and 20% chance of related developmental difficulties (Messinger et al., 2013). Infants are recruited prenatally or in early infancy and assessed at several timepoints until they reach age 2-3 years, when a multidimensional assessment characterises their developmental outcome. Data on early development can be linked to later dimensional and diagnostic outcomes. More recently, such studies have expanded to include infants with genetic syndromes linked to higher autism rates (e.g., tuberous sclerosis, McDonald & Jeste, 2021) and infants with a family history of ADHD (Miller et al., 2020), and have incorporated information about polygenic scores and their association to early neurodevelopment (Gui et al., 2020). Longer-term follow-up of such cohorts into school age have begun, with a focus on later-emerging common co-occurring conditions (e.g., ADHD and anxiety, Shephard et al., 2019). Thus, this field investigates how familial and genetic factors are translated into an autism diagnosis through early brain development.

### Emergence of social and non-social changes

Prospective studies have not supported initial ideas that infants with later autism would show profound social differences from early in infancy. Rather, 6-month-old infants with later autism look similar on vocalisations and attention to faces (Ozonoff et al., 2010), and interest in eyes (Jones & Klin, 2013) to those with a neurotypical outcome. Over the following two years, infants with later autism show a gradual decline in looking to faces and eyes (Gangi et al., 2020). Proposed explanations for this include measurement error (e.g., challenges in identifying manifestations of social difficulties in young infants, as they have limited social capabilities); a ubiquitous regression-like profile, only recognised by parents of children with particularly precocious early skills (Ozonoff & Iosif, 2019); or a failure to shift from subcortical to cortically-mediated social attention (Klin, Shultz, & Jones, 2015).

Restricted and repetitive behaviours emerge on a similar timescale, first becoming measurable around 12 months, although earlier developmental manifestations may have yet to be identified (Ozonoff et al., 2011). Indeed, neural changes appear early in both social and non-social domains (e.g., Lloyd-Fox et al., 2018), suggesting that from its earliest emergence autism is associated with subtle differences across multiple domains of brain development. Initial machine-learning approaches to predicting autism from infant data highlight widespread alterations in cortical thickness (Hazlett et al., 2017), functional brain activity across a range of scalp regions and frequency bands (Gabard-Durnam et al., 2019), and alterations to both social and non-social stimulus-locked processing (Tye et al., 2020). This evidence makes it implausible that a single brain ‘deficit’ solely responsible for the development of autism will emerge.

## 3 Clinical applications of early autism research

### Predicting emergence and outcomes

Although work on the early development of autism has yielded important insights, translation to clinical practice has only just begun. With regard to potential for early identification, some infants begin to display behaviours consistent with a diagnosis from 12 months (Pierce et al., 2019), but many others will not show a clear enough pattern of symptoms until age 2 or later (Ozonoff et al., 2015). Even within prospectively assessed cohorts overseen by experienced clinicians, some children considered to have autism in mid-childhood did not meet autism criteria at age 3 (Ozonoff et al., 2018), which complicates prediction and hence raises a range of ethical issues that we discuss below. Further work on the parameters yielding reliable individual estimates is necessary, but clinical prediction based on single measures is unlikely to be successful if autism represents the accumulation of inherited predispositions that act additively (Constantino, Charman, & Jones, 2021) or interactively (Johnson et al., 2021) to determine outcomes.

In response, some have attempted to identify more biologically homogeneous ‘subtypes’ of autism (Wolfers et al., 2019) that could be associated with distinct early developmental pathways. It could be fruitful to align these approaches with the DSM shift from subgrouping within autism towards subgrouping based on profiles of associated difficulties (e.g., with/without intellectual disability or language delay). Indeed, early infant profiles may be more relevant in predicting constellations of dimensional traits than categorical diagnoses (e.g., cognitive, adaptive skills or autistic traits, Jones et al., 2020). For example, Hendry et al. (2020) identified subgroups of infant sibs by their trajectories of development of attention skills; infants who showed a profile of plateauing attentional growth between 10 and 25 months were more likely to have elevated autism and ADHD traits, and lower adaptive function at 3 years. Longer-term follow-up of such cohorts is required to determine whether such early neurodevelopmental profiles make autism more likely to be diagnosed in early development, rather than represent a subtype of autism that meaningfully persists over developmental time.

### The concept and current practice of pre-emptive intervention

One of the primary stated motivations for identifying early markers of autism is that very early intervention at the time of greater brain plasticity may be especially effective (Webb, Jones, Kelly, & Dawson, 2014). This involves a shift from viewing autism as a categorical state that is determined from birth by genetic factors, to a polygenic and multifactorial condition with a spectrum of possible presentations whose symptoms result from an atypical developmental path that could be targeted with early intervention. As such, some have proposed that early interventions could prevent or ameliorate the emergence of the disabilities associated with autistic traits if successful (Klin et al., 2020). Traditional biomedical perspectives couched this as a ‘preventative approach’, although nowadays a more acceptable concept is that of ‘pre-emptive interventions’ (Insel, 2007). Under our definition the latter are early and prodromal interventions, initiated before the full expression of a condition, that seek to mitigate developmental risk and optimise outcomes. This can include both approaches seeking to ameliorate the emerging onset of manifestations (early symptoms) of autism (closer to the traditional ‘prevention’ notion); and approaches enhancing and supporting compensatory factors or alternative developmental pathways that promote broader developmental competencies and outcomes, which differ from, but may interact with, the unfolding expression of autism traits or symptoms in the individual (closer to a ‘skills’ rather than a ‘deficit’ approach).

Observational research with infant siblings can provide two important classes of insight to designing early intervention programs. First, information about the nature and timing of early developmental delays can inform the design of intervention programmes that target the classes of early emerging symptoms that are manifested in delayed, or atypical onset of, typical developmental skills. The second, and perhaps more fruitful, avenue is to investigate protective or compensatory factors in early development that can be targeted with supportive interventions. This includes supporting interactions between infants and their parents (Wan, Green, & Scott, 2019); or targeting modifying factors (like effortful control/executive functioning) that can interact with earlier neurodevelopmental changes to shape trajectories in a typical or atypical direction (Johnson et al., 2021). Therapies that aim to strengthen these modifying factors (e.g., executive function skills) could act to buffer development towards an optimal outcome for the child.

### The first wave of early intervention studies

A range of parent-mediated interventions have been trialled in infants in the autism context to enhance aspects of social engagement and attention via ‘environmental enrichment’. These focus on enhancing parent-child dyadic communication and engagement (Landa, 2018), but may also increase caregiver knowledge and empowerment and may reduce parental stress. Interventions in infancy are sometimes applied in a ‘selective’ manner to individuals at elevated likelihood of a condition (e.g., infants with a family history of autism), with a focus on general enrichment to mitigate early emerging atypicalities. Alternatively, they have been applied in an ‘indicated’ manner in infants showing very early signs of a condition (e.g., via a screen), with a focus on these emerging symptoms even if they are not yet well established (Green, 2019). For example, Green and colleagues adapted the parent-mediated Video Interaction to Promote Positive Parenting (VIPP) programme and tested its preliminary efficacy in a 12-week pilot randomized controlled trial (RCT) with 10-month-old infants at elevated familial likelihood of autism. At 14 months the intervention group showed increases in parental non-directiveness and infant attentiveness to parent (Green et al., 2015), and at 36 month follow-up an overall reduction in autism traits as measured over time with Autism Observation Scale for Infants (AOSI) and the Autism Diagnostic Observation Schedule (ADOS) (Green et al., 2017). There were no differences on standardised measures of developmental, language or communication skills. Two recent RCTs using an ‘indicated’ design with 12-month-old infants identified via screening are Whitehouse et al. (2019), which reported no differences in parenting behaviour or in child dyadic communication; and Watson et al. (2017), which reported increases in parental responsiveness but no changes in infant adaptive functioning or language. Neither of these studies reported amelioration of early autism traits post-intervention, although both have yet to report on longer-term outcomes.

Two crucial challenges arise from early identification and intervention studies. First, definition of the appropriate target of prediction and intervention, which requires a judgement as to the most important developmental outcomes for children. Second, decision about who should make this judgment. As we discuss below, early autism research gives rise to potentially conflicting rights, priorities, and interests among children, parents, and autistic adults. In the few intervention trials conducted to date, the primary and secondary outcomes are often a combination of proximal developmental ‘precursors’ of later developmental outcomes (e.g., infant attentiveness), more distal developmental outcomes (e.g., language and communication skills), but also measures of early emerging autism symptoms such as the AOSI and the ADOS. It should be noted that early emerging symptoms seem to predict later co-occurring conditions (e.g., anxiety), whose treatment is high among autistic adults’ priorities (Leadbitter, Buckle, Ellis, & Dekker, 2021). However, the appropriate outcomes to measure in early pre-emptive intervention studies may be broader than a reduction in autism traits or symptoms (Kasari, 2019), as these are not present (or not fully substantiated) in the first 12-18 months of life, and intermediate phenotypes at this stage are likely shared between children with later autism and those who will have other neurodevelopmental conditions (Constantino et al., 2021).

From a clinical perspective an approach targeted toward a wider array of developmental competencies that share commonalities with, but are in part distinct from, early autism signs is warranted. Indeed, many young children with broader early emerging neurodevelopmental difficulties struggle to communicate and interact with others; this restricts their opportunities to learn and develop, and affects their parents, who can find their child’s behaviour challenging (Charman, 2019). Additional early neurodevelopmental phenotypes shared by infants who may later develop autism or other neurodevelopmental conditions (e.g., difficulties in early executive attention) might also be amendable to interventions that act trans-diagnostically, and potentially have effects on the later emergence of common co-occurring traits (Talbott & Miller, 2020). Thus, selecting appropriate outcome measures in early intervention studies will require deep knowledge of developmental cascades, in addition to an integration of the perspectives of all those involved. Below we show that, despite the laudable goals of early interventions seeking to optimise children’s outcomes, our increasing understanding of children’s neurodevelopment in very early life raises a number of ethical challenges.

## 4 Overview of the ethical issues

Early identification and intervention in the context of autism pose significant challenges to three premises of ethically-robust clinical interventions: we know what to intervene on; we can reliably assess benefits and harms; and we have good reasons to intervene (Singh, 2016). We present such challenges as organized around the following themes: (1) New models for understanding autism; (2) Risk and benefits of early identification and intervention; and (3) Communicating early concerns to families.

### New models for understanding autism: What could and should we intervene upon?

Research into early neurodevelopment has led to new models for conceptualising autism, with implications for what could and should be targeted in early life. Increasing recognition of the role of heterogeneity and the lack of clear biological and genetic boundaries among different neurodevelopmental conditions, has inspired a shift from the categorical focus more typical of diagnostic manuals like the DSM, to dimensional and other stratification approaches like RDoC or ESSENCE (see Gillberg & Fernell, 2014). The application of these models in autism aligns with increasing acknowledgement of the dimensionality of psychiatric conditions more broadly (Kong & Singh, 2018), and could allow interventions to be more precisely targeted to particular symptom dimensions (like communication problems). However, in clinical setting, psychiatric diagnoses have an important pragmatic and social function (e.g., for access to relevant support and services for individuals and families). Moreover, shifting to a purely dimensional view might impact the identity and sense of self of people diagnosed with autism. Emerging evidence among people previously diagnosed with Asperger’s, who identified with their diagnosis and did not see it as interchangeable with the autism label, suggests that the integration of the Asperger’s diagnosis into the broader ‘autism spectrum disorder’ diagnosis in DSM-5 has had negative impacts on dimensions of self-understanding (Smith & Jones, 2020).

The trend towards dimensional approaches and recognition of substantial biological heterogeneity in the broader field has begun to influence prospective studies of autism emergence. Although often implicit, two conceptual stances can be detected. The first (simpler) view is that dimensional variation in infant phenotypes will map onto dimensional variation in domain-relevant later strengths and difficulties, particularly in the area of conditions commonly associated with autism. For example, dynamic modulation of frontal theta EEG predicts later variation in core cognitive skills (Jones et al., 2020); early infant fearfulness predicts later anxiety; early heightened activity level predicts later ADHD symptoms (Shephard et al., 2019); and infant over-connectivity in the alpha oscillatory EEG band relates to later restricted interests (Haartsen et al., 2019). These insights do raise the possibility that infant interventions could be targeted to particular domains of difficulty that are considered a priority for the autistic community, although currently these patterns are correlational, and causality should not be assumed.

An alternative (or complementary) view is that a separable set of inherited predispositions sum to trigger the emergence of the behavioural symptom clusters that we label as autism (Constantino et al., 2021). This may operate through a whole-brain process in which behavioural traits like repetitive behaviour or social withdrawal represent adaptive reactions to a brain that processes the environment differently (Johnson et al., 2021); or represent the consequences of a lack of necessary early experiences (Klin et al., 2020). In both models, interventions that alter the degree to which an infant experiences these early dimensional traits could impact the likelihood of emergence of later autistic behaviours. However, dimensional variation in the infant predictors of later autism is not necessarily hypothesised to map simply and predictably onto dimensional autistic trait variation in later development, because of the intermediate operation of whole-brain or stochastic processes (Constantino et al., 2021). Thus, it should not be assumed that interventions targeted at particular processes in early infancy would translate in a simple way to predictable shifts in later phenotypes.

Models that view the behavioural traits associated with autism not as maladaptive, but as the result of an *alternative* developmental pathway, or as an *adaptive* response to a brain that processes information in a different way (Johnson, 2017; Johnson, Jones, & Gliga, 2015), accommodate the idea that variation between individuals is a critical part of our adaptive success as a species. This viewpoint is often obscured by biomedical models that focus on individual ‘impairments’ and ‘deficits’ relative to typical development. The ‘deficit’ language, and so assumptions that any difference in an autistic child must be ‘worse’, make it difficult to assume ability, potential, and advantageous traits in autistic children. Defining neurodevelopmental diversity by its disadvantageous elements or core deficits might also preclude the development of interventions whose delivery and success depend on those advantageous traits (Astle & Fletcher-Watson, 2020).

Although this line of thinking is familiar in autism studies, and resonates with the claims of the neurodiversity movement (Kapp, 2020), we argue that adaptive models of autism raise important questions about the feasibility and desirability of the early intervention agenda, which in the existing literature have only been discussed to a limited extent (e.g., Mottron, 2017), and have not been fully addressed in current research practice. First, if autism is the result of the (additive or interactive) accumulation of a range of separate predispositions, early neurodevelopmental markers may not look like later features of autism (Johnson, 2017). This challenges the assumption that we know what we could intervene upon in early infancy, because at this stage the structure of autism could present differently from the autism symptoms that are consolidated in early childhood behaviour. Moreover, if autism ‘symptoms’ are in fact the result of necessary adjustments or responses to an atypical starting state, intervening early on these ‘symptoms’ might have negative implications on other functions they compensate for. This raises the question whether we should intervene early in the development of autism in the first place, or at least makes it ethically compelling to carefully consider the degree to which we understand the system in which we are intervening. However, if we view some of the dimensional traits that lead to autism as making it difficult for a child to learn from the typical environments provided to infants and young children (Klin et al., 2020), it may also be unethical *not* to provide an environment in which autistic children can learn.

### Early identification and interventions: Can we properly weigh risks and benefits for all those involved?

One fundamental challenge of early autism research is that our current ability to predict later autism is (and may remain) probabilistic given the interaction of multiple genetic and environmental factors in shaping trajectories. With any probabilistic outcome, attempts at early prediction may lead to false positives; this can trigger a range of unnecessary surveillance strategies (Wolff & Piven, 2020). Conversely, because early in life infants with later autism show behavioural traits that are similar to those of typically-developing children, risk of false negatives should not be underestimated, especially considering that parents have highlighted the long time lag between first concerns about their child’s development and their age of diagnosis (Szatmari et al., 2016). Furthermore, some have questioned the generalisability of behavioural signs identified in infant sibling design studies (Szatmari et al., 2016), which echoes broader discussions about the appropriateness of universal early screening vs. screening of symptomatic children (Graf, Miller, Epstein, & Rapin, 2017).

Within such a high level of uncertainty, it is difficult to make reliable individual assessments of risks and benefits of early identification and intervention. One option could be to conduct more research to better corroborate early neurodevelopmental markers for individual prediction of categorical autism, so that interventions that are designed to be appropriate for children with autism are not widely applied. At a minimum, these interventions should avoid repeating the history of adopting painful aversives to treat autism, and oppose the current trend of poor monitoring and reporting of adverse events in research on psychosocial interventions for autism (Bottema-Beutel, Crowley, Sandbank, & Woynaroski, 2020). However, the probabilistic nature of predictions means that in early autism research there will always be children who will grow up following a neurotypical profile. Thus, it would also be important to develop interventions targeted to domains of relevance to all children – such as language abilities – to ensure a positive benefit/harm ratio for any child.

It is important to note that uncertainty is not confined to our capacity to predict whether an infant will later develop autism or not; it also extends to our capacity to predict autistic people’s future outcomes from childhood data. While we have stronger confidence in early predictors such as language development, prediction of global outcomes increases through to age 9 years (Pickles, McCauley, Pepa, Huerta, & Lord, 2020); and for outcomes such as mental health, prediction is only possible with data collected in adolescence, or it is not possible at all (Forbes, Lord, Elias, & Pickles, 2021). Acknowledging that very early development *influences*, rather than *determines*, autistic individuals’ future outcomes is fundamental to oppose certain assumptions that may (and do) harm children and their parents. Among these is the assumption that, past 2-3 years of age, it is too late to intervene in a child’s development, which puts excessive pressure on parents to do anything possible during their children’s first few years, and may lead some to argue more strongly for and justify dangerous therapies (e.g., chelation, James, Stevenson, Silove, & Williams, 2015) for children beyond that age. In turn, this has nurtured a research culture which at times allows unnecessarily invasive, painful, and distressing procedures to be performed on young autistic children for the sake of identifying biomarkers for autism (e.g., Pardo et al., 2017); and which places undue emphasis on early intervention at the expense of ensuring support to autistic people throughout the lifespan.

These considerations highlight the importance of assessing risks and benefits of early identification and intervention from different perspectives, by consulting with autistic people, across childhood and into adulthood, as well as their parents. Indeed, early autism research gives rise to two competing ethical principles. On the one hand, there is parents’ right to know about their children’s susceptibility to neurodevelopmental conditions, and their right to intervene on such susceptibility, to act in the best interests of the child or the family more broadly. Parents’ views are central to discussions around pre-emptive interventions, as these involve very young children. On the other hand, some scholarship on corrective or enhancing interventions on children has argued that a child’s ‘right to an open future’ should be respected (Feinberg, 1992). One might argue that ‘right to an open future’ has limited teeth, given that children are inevitably and necessarily the subject of their parents’ influences and interventions from the start. However, the spirit of the ‘open future’ principle is useful when judging which actions of parents might violate ‘best interests’ at a stage when children are wholly dependent on their parents to shape their developmental trajectories. Labelling a child early in life as ‘at risk’ of neurodevelopmental challenges, and so as in need of increased surveillance could lead parents and the wider society to treat the child as someone who *will* eventually develop autism (MacDuffie, Estes, Peay, Pruett, & Wilfond, 2021). Thus, stigmatizing attitudes could be directed towards the child, due to the negative assumptions people often hold about autism. Moreover, autistic adults have criticized certain early interventions, envisioned to make autistic children indistinguishable from their peers, as being motivated by a ‘normalization’ agenda (e.g., Applied Behavioural Analysis, Ne’eman, 2010). The ‘right to an open future’ ideal does not exclude the possibility that interventions can be compatible with neurodiversity interests (Leadbitter et al., 2021). Rather, it calls on us to judge the rightness of interventions on a very young child against a wider range of potential good outcomes for that child, one of which is establishing an authentic sense of self, and flourishing with autism.

### Communicating early concerns about a child’s development to their families: Are concerns widely shared?

In studies of infants with a family history of autism, specific harms might derive from communicating early concerns about a child’s development to parents. With neurodevelopmental markers that remain probabilistic, and in the context of trials that do not always offer an intervention component, raising concerns is recognised as problematic (MacDuffie et al., 2021). It could harm parents, by (perhaps unnecessarily) increasing their anxiety during a period of uncertainty about their child’s future or removing hope about their child’s development.

A related, but less discussed, issue is whether atypical development should be a matter of concern in the first place. A variety of views exist on the value judgments that should be attached to autism, and the proposition that flourishing with autism is both reasonable and desirable is an important motivation for the neurodiversity movement and its allies (Kapp, Gillespie-Lynch, Sherman, & Hutman, 2013). Thus, not everyone in the autism community agrees with certain understandings of ‘optimal’ development that underpin early intervention approaches, particularly those that aim at preventing a diagnosis later in life, and so with the premise that we have good reasons to intervene early in the development of autism to slow down or stop its progress. Some instead favour research that would shed light on the unique development of autistic individuals (Fletcher-Watson et al., 2017). This may be particularly the case in families enrolled in early intervention trials, who likely have enhanced rates of autism traits or diagnosis, including in the parents themselves (MacDuffie et al., 2020), and so may be against pathologizing autism.

Moreover, some of the early intervention literature seems to be grounded on implicit normative assumptions on what being a good parent means (Mortimer, McKeown, & Singh, 2018). Effective parent-mediated interventions require an understanding of what aspects of parenting behaviour are relevant for optimal neurodevelopment, in addition to agreement on the definition of ‘optimal’. Because parenting interventions involve discussions about the influence of parents’ behaviours on their infants’ developmental progress, framing such progress as concerning may be offensive to families who have accepted autism as part of their identity and their family bond. At the same time, early intervention research and programmes might nurture parents’ self-blame for having caused the difficulties their children experience, particularly given the burden of historical accusations, such as the ‘refrigerator mother’ (Bettelheim, 1967). Early autism researchers should continue to be aware of how their research could (even if unintentionally) shape conceptions of the good life, good parenting and good developmental outcomes. Researchers should be supported to anticipate and manage such consequences in their research and in respectful engagements with families.

## 5 Reshaping early autism research

The above discussion shows that important ethical concerns emerge from research on early neurodevelopment in the autism field, and from psychosocial interventions being developed as a result of this research. We propose several recommendations to address these concerns.

First, efforts to identify early neurodevelopmental markers for autism should go together with research investigating autistic people’s attitudes towards new emerging models of autism. In addition, studies should address ethical considerations such as authenticity, flourishing, blame, responsibility and interests, among parents of autistic children enrolled in early intervention studies. Investigations should also address how older autistic siblings perceive efforts to intervene early in the development of autism in their younger siblings, and possible implications on their self-understanding. Such research will be methodologically challenging, particularly given the range of capabilities of autistic children. However, recent methodological advances indicate the potential for eliciting first-person perspectives of autistic young people with minimal verbal abilities (Tesfaye et al., 2019). Finally, longer-term follow-up of cohorts of infant siblings into school age have begun, and it is reasonable to posit a research agenda that investigates the attitudes and first-person experiences of early and pre-emptive interventions among these children. Case studies of families could address ethical questions around the impact of such experiences on younger siblings’ self-perceptions, and on their relationship with their older autistic siblings.

For these themes to be addressed in future studies, early autism research cannot be confined to laboratory work; rather, cross-disciplinary collaborations adopting methods from the basic sciences and the humanities and social sciences are needed. Because we were only able to offer an overview of some of the emerging ethical issues, bioethicists should further interrogate the empirical dimensions of the ethical challenges identified here, as well as identify any gaps in our analysis. Alongside this empirical work, it is also important to conduct further normative assessment of the potential harms and benefits of early identification and intervention, to better inform researchers, parents and policy makers. Autism research is both a model for, and a lesson in, the importance of conducting research with members of a community bound together by a common set of psychiatric or developmental labels. Methods that enable inclusion of the neurodevelopmentally, politically and demographically diverse range of autistic children and adults are particularly important to develop and implement.

## 6 Concluding remarks

Our aim was to summarise the ethical issues emerging from new research on the identification of neurodevelopmental markers for autism. The great majority of ethical analysis to date has focused on genomic markers for autism; however, there are significant and distinctive ethical considerations for psychosocial interventions, affecting infants, parents, other family members and society as a whole. Our analysis has highlighted the importance of a two-way dialogue between early autism researchers and ethicists, to help facilitate research and real-world applications and psychosocial interventions that promote flourishing in children, families and societies. Such an approach requires expertise and methods from the basic sciences and bioethics, as well as constructive collaborations between autistic people, their parents, and autism researchers. Together, we can work towards early interventions that serve the autism community’s interests and accommodate the varied experiences of people on the spectrum and their families.

## Key points

- Early autism research has inspired dimensional models of autism, which might impact on the self-understandings of people diagnosed with autism; and adaptive models, which question the desirability of intervening upon potentially necessary early responses to a brain that processes information differently.
- Early autism research faces methodological challenges around individual prediction of later autism, which challenges reliable risk/benefit assessments of early identification and intervention.
- Early intervention into autism raises potential conflicts between parents’ right to know about their children’s susceptibility to autism and children’s right to an open future.
- Early autism researchers should reflect on whether their research imposes certain conceptions of optimal developmental outcome and/or good parenting.
- Future research should address ethical considerations such as blame and responsibility among parents; investigate older autistic children’s attitudes towards early identification and intervention; and explore the impact of experiences of early interventions on younger siblings.

## Footnotes

1 We use the term ‘autism community’ to refer to a broader group than people with an autism diagnosis or people who self-identify, and to include their family members, particularly their parents and siblings, as well as autism researchers.
